# RETRACTION: Bayesian‐Edge System for Classification and Segmentation of Skin Lesions in Internet of Medical Things

**DOI:** 10.1111/srt.70279

**Published:** 2025-10-21

**RETRACTION**: S. Naseem, M. Anwar, M. Faheem, M. Fayyaz, and M. S. A. Malik, “Bayesian‐Edge System for Classification and Segmentation of Skin Lesions in Internet of Medical Things,” *Skin Research and Technology* 30, no. 8 (2024): e13878, https://doi.org/10.1111/srt.13878.

The above article, published online on 31 July 2024 in Wiley Online Library (wileyonlinelibrary.com), has been retracted by agreement between *Skin Research and Technology*; and John Wiley & Sons Ltd. The article was accepted for publication following an insufficient peer review process that does not meet the standards of the journal's peer review policy. Post‐publication review identified several concerns included, but not limited to, methodological flaws, irrelevant self‐citations, and copyright violation. Accordingly, the article has been retracted. The authors have been informed of the retraction decision and disagree with it.

